# Comparing comparisons: A transdiagnostic investigation of social and temporal comparisons and their differential effects on mental health outcomes and well-being

**DOI:** 10.1016/j.ijchp.2025.100611

**Published:** 2025-07-21

**Authors:** Pascal Schlechter, Dana Churbaji, Thole H. Hoppen, Nexhmedin Morina

**Affiliations:** Institute of Psychology, University of Münster, Germany

**Keywords:** Social comparison, Temporal comparison, Transdiagnostic, Depression, Comparison theory

## Abstract

**Background:**

While the association of social comparisons and mental health has been frequently researched, the role of temporal comparisons (evaluating oneself over time) and their distinct associations with mental health outcomes are understudied. Here, we aimed to elucidate the distinct associations of social and temporal comparisons with a range of mental health outcomes, as well as previously identified predictors of these outcomes. We specifically examined the differential relationships of comparison frequency, discrepancy, and affective impact with depression, anxiety, posttraumatic stress, well-being, life satisfaction, self-esteem, metacognitions, rumination, and self-efficacy.

**Methods:**

To this end, we conducted a thorough reanalysis of data from one longitudinal and five cross-sectional studies sourced from Prolific Researcher with English speaking participants. One of these studies involved participants with elevated depressive symptoms. Additionally, we included one study with Syrian refugees in Germany recruited via social media.

**Results:**

Across the seven studies (*N*s = 306 to 1121), regression models revealed consistent and mainly moderate associations between both social and temporal comparisons and our outcomes. Additionally, our findings suggested only a weak trend for social (vs. temporal) comparisons to exhibit stronger associations with mental health variables.

**Conclusions:**

Our results offer insights into the role of social and temporal comparisons in mental health, providing a foundation for follow-up research that may ultimately inform psychological interventions.*Keywords.* Social comparison, temporal comparison, transdiagnostic, depression, comparison theory.

Comparative thinking is a transdiagnostic factor across mental health-related variables, including depression (McCarthy & Morina, 2020), anxiety (Schlechter, Hellmann et al., 2024), self-esteem (Schlechter, Katenhusen et al., 2023), posttraumatic stress disorder (PTSD; Hoppen et al., 2020), metacognitions about worries (Schlechter, Hellmann et al., 2024), rumination (Schlechter, König et al., 2023), social anxiety (Goodman et al., 2021), body dissatisfaction (Fitzsimmons-Craft et al., 2015), self-efficacy (Churbaji & Morina, 2024), and psychological well-being (Morina & Schlechter, 2023). Social comparison (Festinger, 1954) - evaluating oneself in relation to others - is by far the most studied comparison standard (Gerber et al., 2018). However, individuals also frequently engage in temporal comparison (Albert, 1977) through evaluations of their present selves in relation to their past or anticipated future selves, with potential implications for their mental health. Overall, temporal comparisons are as frequently reported as social comparisons (Wilson & Shanahan, 2020). However, it has been argued that individuals may be overly reliant on social rather than temporal comparison in their evaluation of their performance (Van Yperen & Leander, 2014). Our research aims to elucidate the relative contributions of social and temporal comparisons to various mental health outcomes, given the lack of research on their differential associations with mental health outcomes.

The temporal comparison theory (Albert, 1977) and the general comparative-processing model (gComp; Morina, 2021) outline conceptual parallels between social and temporal comparisons. Broadly, comparisons can be categorized into upward (better-off), downward (worse-off), or lateral (equivalent) standards (Gerber et al., 2018). Comparisons perceived as posing a threat to the comparer’s self-motives are defined as aversive (Morina, 2021). Theoretically, upward social (e.g., *doing worse than other individuals*), past temporal (e.g., *I used to be doing better than now*), and downward prospective temporal (e.g., *I will be doing worse in the future*) comparisons are defined as aversive (Morina & Schlechter, 2023). The comparison process starts with the selection of a standard within a specific dimension, for instance one’s well-being or appearance. Then, individuals evaluate the (dis)similarities between their self-attribute and that of the chosen standard. The result represents the perceived *discrepancy* between the comparer’s well-being or appearance and the standard. The comparison may then change the comparer’s affect relative to their baseline, which is defined as the *engendered affective impact.*

Despite their parallels, social and temporal comparisons may be differentially associated with both mental health outcomes and their established predictors. Social comparisons have been associated with various mental disorders, including social anxiety disorder (Goodman et al., 2021), body dysmorphic disorder (Anson et al., 2015), depressive disorders (McCarthy & Morina, 2020), and eating disorders (Corning et al., 2006). Individuals suffering from these disorders compare more frequently to others, which may contribute to the maintenance of psychopathology. Conversely, the limited evidence on temporal comparisons points to an association among temporal comparisons and PTSD symptoms in traumatized populations (Hoppen et al., 2020). In addition, studies examining temporal comparisons in concert with social and other comparison types have linked them to various mental health outcomes such as symptoms of depression (Morina & Schlechter, 2023), anxiety (Schlechter, Hellmann et al., 2024), and PTSD (Schlechter, Hoppen et al., 2024). However, an examination of the unique associations of social vs. temporal comparison was not conducted in these studies. Experimental studies suggest that in a performance context, individuals rely on social comparisons over temporal comparisons, and some authors have concluded that there is an overpowering effect of social comparison (Van Yperen & Leander, 2014). In three experimental studies where participants were provided false social and temporal feedback about their cognitive performance, a dominant reliance on social comparison over temporal comparison was observed (Van Yperen & Leander, 2014). However, a comprehensive investigation into the unique associations of social vs. temporal comparisons with mental health outcomes is lacking. This accounts especially for the components of the comparison process outlined in gComp (i.e., comparison frequency, discrepancy, and affective impact, Morina, 2021). Beyond understanding the impact of social and temporal comparisons on mental health outcomes, it is crucial to explore their links to established mental health predictors, including self-esteem (Orth et al., 2018), metacognition about worries (Wells & Cartwright-Hatton, 2004), rumination (Ehring & Watkins, 2008), life stressors (Churbaji & Morina, 2024), or self-efficacy (Xing et al., 2023). Analyzing these associations can reveal relevant relationships and advance theoretical models that can be tested in future research. For example, if research shows that temporal comparisons, but not social comparisons, are strongly correlated with metacognitive beliefs, future studies can focus specifically on this relationship to reveal distinct pathways towards anxiety or depression. Understanding the unique relationships of social and temporal comparison components with variations in mental health outcomes and their predictors is crucial for advancing our understanding of cognitive and emotional processes underlying specific mental disorders.

In this project, we investigated the differential associations of social vs. temporal comparisons (comparison frequency, discrepancy, and affective impact) on 1) a range of mental health variables and 2) relevant transdiagnostic predictors of mental health outcomes. Specifically, we reanalyzed data from seven existing studies that examined comparative thinking in the context of appearance and well-being in relation to mental health. While these studies have examined the shared influence of aversive comparisons, they have not investigated the unique impact of social vs. temporal comparisons. First, we aimed to discern the specific relationships of social vs. temporal comparison with mental health outcomes, including depression, anxiety, PTSD symptoms, psychological well-being, and life satisfaction. Second, we investigated their differential associations with established predictors of mental health outcomes, namely self-esteem, self-efficacy, metacognitions about worries, brooding rumination, and postmigration stressors. Table 1 outlines how social and temporal comparisons may be associated with mental health outcomes and mental health predictor variables. We expected both social and temporal comparisons to be at least moderately related to our outcome variables. Yet, given the limited prior research on social vs. temporal comparison and the outcome selection being guided by available data, we did not formulate a-priori hypotheses regarding their differential impact on specific mental health outcomes.

**Table 1:** Overview of the assessed constructs and their hypothesized relationship with aversive social and temporal comparisons on the well-being dimension.

**Construct**	**Social Comparisons** Frequent comparisons of one’s own well-being to those of others who seem to be doing better may be mutually related to:	**Temporal Comparisons** Frequently reflecting on how one used to be doing better in the past or that one will be doing worse in the future may be mutually related to:
**Mental Health Outcomes**		
*Depression*	feeling inadequate, hopeless, sad, lowered motivation, and a lack of self-worth in the light of negative self-evaluations (McCarthy & Morina, 2020).	
*Anxiety*	worry about anticipated negative evaluations of others and what future might bring (Schlechter, Hellmann et al., 2024).	
*Life Satisfaction / Well-being*	lower life satisfaction and psychological well-being (Schlechter & Morina, 2024).	
*PTSD*	self-blame, especially if the comparer perceives others as being more resilient than themselves, which may exacerbate symptoms (Morina, 2020).	
**Predictor Variables**		
*Self-esteem*	lower self-esteem, an important transdiagnostic variable across several mental disorders (Orth et al., 2018).	
*Metacognitions*	a cycle of persistent worry, negative metacognitions about worrying, and anxiety. These metacognitions can contribute to chronic stress and anxiety (Wells & Cartwright-Hatton, 2004).	
*Brooding*	a pattern of negative thinking including brooding rumination, a sense of loss or regret. Brooding constitutes and important transdiagnostic variable (Ehring & Watkins, 2008)	
*Post-migration stressors*	stress that is ultimately associated with a range of mental health outcomes (Malm et al., 2020).	
*Self-efficacy*	a reduced belief in one’s own capabilities and thus more mental health problems (Churbaji & Morina, 2024).	

## Methods

### Openness and transparency

We conducted secondary data analyses from seven studies on comparisons and mental health. While the present research question has not been previously addressed, results of the previous publications indicated that all outcome variables we use display relevant relationships with aversive appearance and well-being comparisons. Sample size considerations were made in the context of the specific research questions of the previous studies. Given our exploratory approach in the context of existing data, none of the studies or procedures was preregistered. Analyses were performed in R (R Core Team, 2021) version 4.3.0. We provide all data, materials, and R codes in the open science framework: https://osf.io/nxwjh/?view_only=5484a0ad75df482eb428355fbf197634.

### Participants and design

The *N*s of the seven different studies ranged from 306 to 1121, including one longitudinal study, one study with participants with elevated depressive symptoms, and one Syrian refugee sample. Studies 1 and 2 assessed appearance as comparison dimension, whereas studies 3–7 examined well-being as comparison dimension. In studies 1–6, participants were recruited via online panel provider prolific (Palan & Schitter, 2018). Prolific is headquarted in the UK and disseminates surveys to adult participants (18 years or older) from most of the 38 OECD (Organization for Economic Cooperation and Development; www.oecd.org) countries. Research indicates that data obtained from prolific have good quality (Douglas et al., 2023). Participants were compensated an hourly reward of at least £6. All studies except Study 3 were cross-sectional studies. In all studies except Study 5 and 7, we recruited participants of the general population who were fluent in English and at least 18 years old. Study 5 was an exception, as we specifically recruited participants with elevated depressive symptoms. An exception in terms of recruitment was Study 7 that used social media platforms to recruit a Syrian refugee sample (e.g., Facebook groups like “Syrian refugees Germany”, Churbaji & Morina, 2024). Ethical approval for all studies was granted by the ethics committee of the Faculty of Psychology and Sports Science the University of Münster. Participants provided informed consent prior to participation, including agreement to the use and reuse of their anonymized data for research.To ensure data quality in all studies, participants needed to pass two out of three attention checks that were implemented in the surveys. We further examined data for careless and/or inconsistent responding (Curran, 2016; Ward & Meade, 2023) using the *Careless* package in R (Yentes & Wilhelm, 2021). For instance, we investigated whether participants had no variance in their data. Also, response times were checked and participants who were exceptionally fast or slow were excluded.

Table 2 shows detailed demographic characteristics of all seven samples. In Study 1 (*N* = 1121), the primary aim was to validate the Comparison Standards Scale for Appearance (CSS-A, see below; Morina et al., 2023). In Study 2 (*N* = 550), a scale concerning attitudes towards social comparison was validated (Schlechter, Meyer et al., 2024). Studies 3 and 4 were conducted within the same overarching research project as Study 2. Study 3 is the only longitudinal study with two time points three months apart consisting of *N* = 1084 participants at the first timepoint and *N* = 942 at the second timepoint (86.9 % of the initial sample). Participants who did not participate (vs. did participate) in the follow-up of Study 3 were significantly more likely to be male (*p* = .004) and younger (*p* < .001). In Study 4, *N* = 306 participants were recruited. In study 5, the Comparison Standards Scale for Well-Being was validated (CSS-W, see below; Morina & Schlechter, 2023). In this study, participants had to score ≥ 5 on the Patient Health Questionaire for depression (PHQ-8; see below), as this is indicative of mild depressive symptoms (Kroenke et al., 2009). In Study 6, *N* = 596 participants participated in an examination of comparative thinking and affective styles (Schlechter & Morina, 2024). In Study 7, *N* = 1070 Arabic speaking migrants who were forcibly displaced were recruited to investigate cognitive factors influencing the impact of postmigration stressors on subjective well-being (Churbaji & Morina, 2024). The present investigation exclusively focuses on the *N* = 991 Syrian refugees in this sample.

**Table 2:** Study and demographic characteristics for all seven studies.

**Study Characteristic**	Study 1 (*N* = 1121)	Study 2 (*N* = 550)	Study 3 (*N* = 1084)	Study 4 (*N* = 306)	Study 5 (*N* = 500)	Study 6 (*N* = 596)	Study 7 (*N* = 991)
**Study design**	Online Prolific cross-sectional study (Morina et al., 2023).	Online Prolific cross-sectional study (Schlechter, Meyer et al., 2024).	Online Prolific longitudinal study, two time-points three months apart (Schlechter, Meyer et al., 2024).	Online Prolific cross-sectional study (Schlechter, Meyer et al., 2024).	Online Prolific cross-sectional study (Morina & Schlechter, 2023).	Online Prolific cross-sectional study (Schlechter & Morina, 2024).	Online cross-sectional study disseminated via social media (Churbaji & Morina, 2024).
**Population/ Sample**	General Population English-speaking population from OECD countries	General Population English-speaking population from OECD countries	General Population English-speaking population from OECD countries	General Population English-speaking population from OECD countries	Participants with mild depressive symptoms English-speaking population from OECD countries	General Population English-speaking population from OECD countries	Syrian refugees with a history of forced migration to Germany
**Comparison** **Dimension**	Appearance	Appearance	Well-Being	Well-Being	Well-Being	Well-Being	Well-Being
**Demographic characteristics**							
Female gender	*n* = 484 (43.2 %)	*n* = 177 (32.2 %)	*n* = 436 (39.4 %)	*n=* 232 (75.8 %)	*n=* 233 (46.6 %)	*n=* 441 (74.0 %)	*n=* 609 (61.5 %)
							
Mean age in years	28.7 (*SD* = 9.7)	33.97 (*SD* = 11.54)	31.56 (*SD* = 10.49)	24.97 (*SD* = 5.93)	28.08 (*SD* = 8.7)	24.83 (*SD* = 7.15)	30.25 (*SD* = 8.50)
Social ladder (1–10)	–	–	5.48 (*SD* = 1.56)	–	–	–	–
Education							
Less than high school	*n* = 13 (1.2 %)	*n* = 6 (1.1 %)	*n* = 11 (1.0 %)	*–*	*n* = 5 (1.0 %)	*n* = 9 (1.5 %)	*n=* 98 (9.9 %)
High school degree	*n* = 268 (23.9 %)	*n* = 92 (16.7 %)	*n* = 198 (18.7 %)	*n* = 148 (48.4 %)	*n* = 123 (24.6 %)	*n* = 204 (34.2 %)	*n=* 335 (33.8 %)
Some college education	*n* = 229 (20.4 %)	*n* = 104 (18.9 %)	*n* = 204 (18.2 %)	*–*	*n* = 103 (20.6 %)	*n* = 126 (21.1 %)	*–*
Associate degree	*n* = 51 (4.5 %)	*n* = 16 (2.9 %)	*n* = 51 (4.7 %)	*–*	*n* = 20 (4.0 %)	*n* = 18 (3.0 %)	*–*
Bachelor's degree	*n* = 358 (31.9 %)	*n* = 234 (42.5 %)	*n* = 417 (38.5 %)	*–*	*n* = 161 (32.2 %)	*n* = 158 (26.5 %)	*–*
Graduate degree	*n* = 202 (18.0 %)	*n* = 98 (17.8 %)	*n* = 203 (18.7 %)	*–*	*n* = 88 (17.6 %)	*n* = 81 (13.6 %)	*–*
University degree	*–*	–	*–*	*n* = 152 (49.7 %)	*–*	*–*	*n=* 558 (56.3 %)
Other	*–*	–	*–*	*n* = 6 (1.9 %)	*–*	*–*	*–*
Marital Status							
Married	*n=345* (30.1 %)	*n* = 202 (36.7 %)	*n* = 372 (34.3 %)	*–*	*n* = 148 (29.6 %)	*n* = 126 (21.1 %)	
Widowed	*n=2* (<1.0 %)	*n* = 4 (<1.0 %)	*n* = 6 (1.0 %)	*–*	*n* = 0 (0 %)	*n* = 0 (0 %)	
Divorced	*n=10* (1.0 %)	*n* = 11 (2.0 %)	*n* = 25 (2.3 %)	*–*	*n* = 2 (<1.0 %)	*n* = 4 (<1.0 %)	
Separated	*n=12* (1.1 %)	*n* = 2 (<1.0 %)	*n* = 5 (1.0 %)	*–*	*n* = 4 (<1.0 %)	*n* = 0 (0 %)	
Single/never married	*n=752* (67.1 %)	*n* = 331 (60.1 %)	*n* = 676 (62.4 %)	*–*	*n* = 346 (69.2 %)	*n* = 466 (78.2 %)	
Migration Status							
Asylum							*n=* 546 (55.1 %)
Family reunification							*n=* 255 (25.7 %)
Visa Application							*n=* 131 (13.2 %)
NHCR Program							*n=* 8 (0.8 %)
Other							*n=* 51 (5.2 %)

## Materials

**Social and temporal comparison frequency, discrepancy, and affective impact** were assessed in all studies**.** In Studies 1 and 2, the CSS-A was used to assess the degree of engagement in upward and downward comparisons via social and temporal comparison standards with respect to one’s own *appearance* during the past three weeks (Morina et al., 2023). The original scale consists of 48 items pertaining to comparison frequency, discrepancy, and affective impact for a range of different comparison types, including social and temporal comparisons. The CSS-A comprises aversive and appetitive (i.e., comparisons that are consistent with or supportive of the comparer’s motives) comparisons. For aversive social comparison, we combined the two following items: “*Over the past three weeks when considering your appearance, how often have you compared with others in your close circles who look better than you?”* and *“…compared with other individuals known and unknown to you who look better than you?”.* For aversive temporal comparison, we combined the two following items: *“… thought that you used to look better than currently?”* and *“… thought that you might look worse in the future than currently?”.* In the present study, we only used the two aversive social and temporal comparison items, as aversive comparison and not appetitive comparison were related with mental health outcomes in previous work. The CSS-A first assesses a) comparison frequency during the past three weeks using a six-point Likert scale (0 = *not at all* to 5 = *very often*). Then, subsequent items ask about b) the perceived discrepancy between target and standard on a six-point Likert scale (0 = *not at all* to 5 = *much better/worse*), and c) the engendered affective impact of the given comparison type on a bipolar seven-point Likert scale (−3 = *much worse* to +3 = *much better*). If participants reported engagement in the respective comparison type, they were then asked “*How much better have you considered their appearance to be?”* (i.e., discrepancy) and “*On average during the past three weeks, how did the comparison make you feel?*” (i.e., affective impact). As such, the scale yields six total scores measuring three aversive comparison process components per comparison type, i.e. social comparison frequency, discrepancy, and affective impact, as well as temporal comparison frequency, discrepancy, and affective impact. In Studies 3–7, well-being comparisons were assessed with the Comparison Standards Scale for Well-Being (CSS-W; Morina & Schlechter, 2023), which mirrors the CSS-A in its structure. In Study 6, only comparison frequency and affective impact were assessed but not comparison discrepancy. In Study 7, we used the Arabic translation of the CSS-W, which displayed good psychometric properties in a previous study (for translation and validation, see Churbaji & Morina, 2024). In Study 7, only comparison frequency was assessed. Internal consistencies (α, ω) ranged from 0.67 to 0.78 for the social comparison subscales, and between 0.48 and 0.58 for the temporal comparison subscales.

## Mental health outcomes

**Depression.** Depressive symptoms were assessed with the Patient Health Questionnaire (PHQ; Löwe et al., 2010). In Studies 1 and 5, we used the 8-item version of the PHQ (PHQ-8), which measures depressive symptoms experienced over the last two weeks on a four-point Likert scale (0 = *not at all* to 3 = *nearly every day*). A total score of ≥ 5 is indicative of at least mild depressive symptoms (Kroenke et al., 2009), which was used as a cut-off for inclusion in Study 5, as we aimed for a sample of participants with elevated depressive symptoms. The internal consistency of PHQ-8 was α = 0.82 in both studies. In Studies 2, 3, 4, and 6, depressive symptoms were assessed using the four-item version of the PHQ (PHQ-4; Kroenke et al., 2009). Two items of the PHQ-4 capture the two hallmark symptoms of depression, *loss of interest and feeling depressed*, on the same four-point Likert scale (0 = *not at all* to 3 = *nearly every day*). Across the six studies, internal consistencies ranged from α = 0.82 - α = 0.85/ ω = 0.83 - ω = 0.86.

**Anxiety.** In Studies 2, 3, 4, and 6, we used the other two items of the PHQ-4 (Kroenke et al., 2009) to assess anxiety on a four-point Likert scale (0 = *not at all* to 3 = *nearly every day*). Items ask participants about the frequency of *feeling nervous* and *not being able to stop worrying.* Internal consistencies ranged from α = 0.82 - α = 0.86/ ω = 0.83 - ω = 0.86.

**Psychological Well-being.** In Studies 1, 5, and 6, we assessed psychological well-being with the 18-item Scale for Psychological Well-being (SPWB, Ryff & Keyes, 1995; e.g., “*In general, I feel I am in charge of the situation in which I live.*”). Items were assessed with a 6-point scale (1 = *strongly disagree* to 6 = *strongly agree*). We used one total score to examine psychological well-being. Cronbach’s alpha ranged from 0.81 to 0.83, while omegas ranged from ω = 0.81 - ω = 0.84. In Study 7, we used the Arabic version of the Well-being Index by the World Health Organization (WHO-5; Sibai et al., 2009, e.g., “*I have felt cheerful and in good spirits”)*. The WHO-5 evaluates overall subjective well-being over the past two weeks using five items on a 6-point Likert scale ranging from 0 = *at no time* to 5 = *all of the time*. Internal consistency was α = 0.90, ω = 0.90.

**Life Satisfaction.** In the longitudinal sample (Study 3), we applied the Satisfaction with Life Scale (SWLS; Diener et al., 1985**,** “*I am satisfied with my life*”), a reliable and valid instrument concerning the overall perception of life satisfaction. It consists of five items on a 7-point Likert scale ranging from 1 = *strongly disagree* to 7 = *strongly agree*. Internal consistencies were good for the first timepoint (α = 0.90, ω = 0.91) and the second timepoint (α = 0.91, ω = 0.91).

**PTSD.** In the longitudinal sample (Study 3), we applied at both assessment timepoints the International Trauma Questionnaire (ITQ; Cloitre et al., 2018) to assess PTSD symptoms. In Study 7, we used the validated Arabic version of the ITQ (Vallières et al., 2018). The ITQ starts with identifying the respondent’s index traumatic event and proceeds with six items assessing PTSD symptoms as defined in the 11th version of the International Classification of Diseases (ICD-11; World Health Organization, 2018). The level of stress caused by these symptoms during the last month is evaluated on a 5-point scale (0 = *not at all* to 4 = *extremely*”). Internal consistencies for the two timepoints were α = 0.86 and 0.85/ ω = 83 ω = 86.

## Mental health predictor variables

**Self-esteem.** In Studies 1, 3, and 4, we assessed self-esteem with the Rosenberg Self-Esteem Scale (RSES, Rosenberg, 1965, e.g., “*On the whole, I am satisfied with myself*”). Items are rated on a 4-point Likert scale (0 = *strongly disagree* to 3 = *strongly agree*). Across studies, internal consistencies ranged from α = 0.91 to α = 0.93/ ω = 0.91 to ω = 94.

**Metacognitions.** In the longitudinal sample (Study 3), we used the beliefs about uncontrollability of thoughts and corresponding danger subscale of the Meta-Cognitions Questionnaire-30 (MCQ-30; Wells & Cartwright-Hatton, 2004; e.g., “*My worrying is dangerous for me*“). Items are assessed on a 4-point scale (1 = *do not agree* to 4 = *agree very much*)*.* Internal consistencies were α = 0.91/ ω = 0.91 at both timepoints.

**Brooding.** In Study 5, we used the Brooding subscale of the Response Style Questionnaire (RSQ; Nolen-Hoeksema, 1991) to assess brooding rumination. This five-item scale instructs participants to report how they typically react to depressed mood on a scale from 0 = *never* to 3 = *always*. Internal consistency was α = 0.64/ ω = 0.65.

**Postmigration Stress.** In Study 7, we used the Refugee Postmigration Stress Scale (RPMS; Malm et al., 2020) which consists of 21 items that evaluate different domains of Postmigration Stress. Response options of this 5-point Likert scale range from 1 = *never* to 5 = *very often*. Participants were for example asked whether they experienced “*discrimination in school or at work”.* Internal consistency was α = 0.83/ ω = 0.84.

**Self-Efficacy.** In Study 7, the General Self-Efficacy Scale (GSES; Schwarzer & Jerusalem, 1995) assessed participants’ confidence in their personal abilities to overcome challenges. The GSES consists of 10 items scored on a 4-point Likert scale, ranging from 1 = *not true at all* to 4 = *exactly true*. We used the validated Arabic version of the scale (Scholz et al., 2002), which had an internal consistency of α = 0.90/ ω = 0.91 in Study 7.

## Analysis

To address our main research questions, we conducted regression analyses. Supplemental Table 1 provides an overview of the skewness and kurtosis of all variables. Skewness was below 1 and Kurtosis below 2 for all variables, thus indicating no substantive deviations from normality (Hair et al., 2006). Moreover, simulation studies demonstrate that correlations stabilize with a sample size around 250 even for slightly non-normal distributed variables (Schönbrodt & Perugini, 2013). All our seven studies had a larger sample size than 300. First, we aimed to discern whether there is sufficient unique variance in the social and temporal comparison standards for our subsequent planned analyses. To this end, we calculated Pearson correlations among the frequency, discrepancy, and affective impact components of aversive social and temporal comparisons. To gauge whether both comparison types are related to a broad range of mental health outcomes, we ran multiple regression models with social and temporal comparison frequency, discrepancy, and affective impact as predictors of the mental health variables (depression, anxiety, psychological well-being, life satisfaction, self-esteem, PTSD, metacognitions about worries, brooding, postmigration stressors, and self-efficacy). To investigate whether social and temporal comparisons differ in their associations with mental health outcomes, we conducted a series of multiple regression analyses using z-standardized predictors to examine the unique contributions of social and temporal comparison processes to our outcomes. In separate analyses, frequency, discrepancy, or affective impact of social and temporal comparisons were entered as the primary predictor. Specifically, we ran three sets of regressions: one with social and temporal comparison frequency, another with social and temporal comparison discrepancy, and a final set including comparison affective impact. Our aim was to test whether social and temporal comparison variables each accounted for unique variance in our criterion variables, which included depression, anxiety, psychological well-being, life satisfaction, self-esteem, PTSD symptoms, metacognitions about worry, brooding, postmigration stressors, and self-efficacy. We descriptively examined the (in)consistency of patterns across studies 1–7, mental health outcomes (are specific patterns only apparent for depression but not anxiety?), and across the components of the comparison process (are specific patterns only evident for comparison frequency but not affective impact?). To reduce the risk of type I error inflation in light of the high number of comparisons, we set the alpha level to 0.001.

## Results

Table 3 shows the descriptive statistics of all constructs for all studies and the correlations among the comparison components (frequency, discrepancy, and affective impact) of social and temporal comparisons. Across the seven studies, 70.6 % (Study 7) to 94.0 % (Study 6) of participants reported at least one social comparison during the last 3 weeks. This compared to 73.1 % (Study 3 T1) to 83.2 % (Study 1) of participants reporting at least one temporal comparison during the last 3 weeks. Correlations ranged from 0.24 [99.9 % CI, 0.15; 0.33] for social and temporal comparisons discrepancy in Study 1 to 0.54 [99.9 % CI, 0.44; 0.63] for social and temporal comparisons affective impact in Study 2. There was thus sufficient unexplained variance in the comparison types for our subsequent analyses.

**Table 3:** Correlations among social and temporal comparison components and descriptive statistics of all measured variables.

	Study 1 (*N* = 1121)	Study 2 (*N* = 550)	Study 3 T1 (*N* = 1084)	Study 3 T2 (*N* = 942)	Study 4 (*N* = 306)	Study 5 (*N* = 500)	Study 6 (*N* = 596)	Study 7 (*N* = 991)
**Correlations among social and temporal comparisons**	r	r	r	r	r	r	r	r
Comparison frequency	.41 [.32; 0.49]	.45 [.33; 0.55]	.49 [.41; 0.56]	.45 [.36; 0.53]	.45 [.29; 0.59]	.34 [.20; 0.46]	.30 [.18; 0.42]	.29 [.19; 0.38]
Comparison discrepancy	.24 [.15; 0.33]	.31 [.24; 0.48]	.36 [.27; 0.45]	.41 [.32; 0.50]	.43 [.26; 0.57]	.27 [.13; 0.40]	–	–
Comparison affective impact	.54 [.46; 0.60]	.54 [.44; 0.63]	.39 [.30; 0.47]	.43 [.34; 0.52]	.38 [.20; 0.53]	.50 [.38; 0.60]	.48 [.37; 0.57]	–
**Descriptive Statistics**	*M* (*SD*)	*M* (*SD*)	*M* (*SD*)	*M* (*SD*)	*M* (*SD*)	*M* (*SD*)	*M* (*SD*)	*M* (*SD*)
*Comparison Process*								
Frequency Social	2.67 (1.39)	2.30 (1.43)	2.45 (1.47)	2.45 (1.42)	2.39 (1.28)	2.41 (1.48)	2.99 (1.26)	1.93 (1.57)
Frequency Temporal	2.27 (1.38)	2.27 (1.43)	1.96 (1.42)	1.97 (1.40)	2.01 (1.35)	1.98 (1.22)	2.11 (1.22)	1.89 (1.31)
Discrepancy Social	2.29 (1.39)	2.52 (1.50)	2.82 (1.54)	2.80 (1.44)	2.47 (1.22)	2.82 (1.54)	–	–
Discrepancy Temporal	1.96 (1.16)	2.31 (1.39)	1.93 (1.33)	1.95 (1.36)	1.93 (1.21)	1.84 (1.18)	–	–
Affective Impact Social	−0.75 (1.06)	−0.70 (1.06)	−0.80 (0.98)	−0.69 (0.99)	−0.60 (0.81)	−1.12 (1.14)	−1.07 (1.12)	–
Affective Impact Temporal	−0.65 (1.05)	−0.73 (1.03)	−0.57 (1.00)	−0.47 (1.01)	−0.42 (0.90)	−0.85 (1.14)	−0.76 (1.15)	–
*Mental Health Outcomes*								
Depression	1.07 (0.74)	0.92 (0.91)	1.06 (0.93)	1.01 (0.93)	0.99 (0.82)	1.41 (0.66)	1.20 (0.98)	–
Anxiety	–	1.02 (0.94)	1.11 (0.91)	1.04 (0.94)	1.16 (0.81)	–	1.40 (0.97)	–
Psychological Well-Being*	3.26 (0.89)	–	3.72 (1.52)	3.91 (1.55)	–	4.34 (0.91)	5.08 (0.84)	3.01 (1.06)
PTSD	–	–	1.61 (1.03)	1.53 (1.03)	–	–	–	1.50 (0.91)
*Predictor Variables*								
Self-esteem	2.26 (0.64)	–	2.73 (0.67)	2.81 (0.66)	3.07 (0.64)	–	–	–
Metacognitions worries	–	–	2.28 (0.90)	2.14 (0.90)	–	–	–	–
Brooding	–	–	–	–	–	2.64 (0.61)	–	–
Postmigration stressors	–	–	–	–	–	–	–	2.85 (0.61)
Self-efficacy	–	–	–	–	–	–	–	2.71 (0.53)

*Note***.***r* = Pearson correlation coefficient, numbers in brackets represent 99.9 % confidence intervals; *M* = mean, SD = standard deviations.

⁎ including life satisfaction.

### Associations with mental health outcomes

Table 4 depicts the regression models examining social and temporal comparison components as predictors of the mental health outcome variables. For depressive symptoms, regression estimates ranged from −0.05 for temporal comparison discrepancy to −0.58 for temporal comparison affective impact. Out of the 52 regression estimates for depressive symptoms (i.e., 26 comparisons of regression estimates), only two estimates for temporal comparison discrepancy and one estimate for temporal comparison affective impact were non-significant, while all effects of social comparison were significant. For anxiety symptoms, regression estimates ranged from 0.04 for temporal comparison discrepancy to −0.57 for social comparison affective impact. Out of the 40 estimates (i.e., 20 comparisons of regression estimates), for five estimates, the estimate of one comparison standard was not significant. In these cases, social comparison frequency (1x), discrepancy 2x), and affective impact (2x) had significant estimates, while temporal comparison frequency, discrepancy, and affective impact were non-significant. For psychological well-being and life satisfaction, regression estimates ranged from 0.07 for temporal comparison discrepancy to 0.52 for social comparison affective impact. Out of the 42 estimates (i.e., 21 regression models), the estimate of temporal comparison discrepancy was non-significant in one model.For PTSD symptoms, regression estimates ranged from 0.05 for temporal comparison affective impact to 0.83 for social comparison frequency. In the 13 regression models, for six estimates, temporal comparison discrepancy (3x) and comparison affective impact (3x) were non-significant.

**Table 4:** Regression results.

	Frequency Social	Frequency Temporal	Discrepancy Social	Discrepancy Temporal	Affective Impact Social	Affective Impact Temporal
***Mental Health Outcomes***						
**Depression**						
Study 1	0.20***	0.17***	0.23***	0.05	−0.24***	−0.13***
Study 2	0.22***	0.13***	0.27***	0.07	−0.27***	−0.08
Study 3 (cross-sectional t1)	0.26***	0.30***	0.23***	0.29***	−0.27***	−0.21***
Study 3 (cross-sectional t2)	0.29***	0.27***	0.31***	0.24***	−0.32***	−0.19***
Study 3 (comp. predicting t2)	0.22***	0.24***	0.22***	0.21***	−0.26***	−0.15***
Depression Study 4	0.16***	0.35***	0.22***	0.30***	−0.24***	−0.24***
Depression Study 5	0.24***	0.17***	0.22***	0.14***	−0.18***	−0.18***
Depression Study 6	0.40***	0.73***	–	–	−0.50***	−0.58***
**Anxiety**						
Study 2	0.29***	0.06	0.28***	0.04	−0.30***	−0.06
Study 3 (cross-sectional t1)	0.30***	0.23***	0.25***	0.24***	−0.31***	−0.12***
Study 3 (cross-sectional t2)	0.35***	0.22***	0.32***	0.20***	−0.34***	−0.17***
Study 3 (comp. predicting t2)	0.29***	0.21***	0.23***	0.21***	−0.28***	−0.15***
Anxiety Study 4	0.19***	0.16***	0.21***	0.12	−0.20***	−0.14
Anxiety Study 6	0.48***	0.53***	–	–	−0.57***	−0.44***
**Psychological well-being/Life satisfaction**						
Study 1	−0.15***	−0.19***	−0.21***	0.07	0.22***	−0.12***
Study 3 (cross-sectional t1)	−0.37***	−0.36***	−0.44***	0.36***	0.52***	0.40***
Study 3 (cross-sectional t2)	−0.32***	−0.43***	−0.42***	−0.40***	0.48***	0.45***
Study 3 (comp. predicting t2)	−0.30***	−0.33***	−0.36***	−0.32***	0.45***	0.36***
Study 5	−0.14***	−0.18***	−0.26***	−0.15***	0.17***	0.24***
Study 6	−0.18***	−0.29***	–	–	0.17***	0.21***
Study 7	−0.21***	−0.24***	–	–	–	–
**PTSD**						
Study 3 (cross-sectional t1)	0.34***	0.14***	0.29***	0.10	−0.27***	0.05
Study 3 (cross-sectional t2)	0.34***	0.15***	0.27***	0.10	−0.29***	0.05
Study 3 (comp. predicting t2)	0.32***	0.18***	0.26***	0.13	−0.35***	0.06
Study 7	0.83***	0.76***	–	–	–	–
***Predictor Variables***						
**Self-esteem**						
Study 1	−0.19***	−0.14***	−0.24***	−0.03	0.24***	0.11***
Study 3 (cross-sectional t1)	−0.27	−0.18***	−0.21***	−0.20***	0.24***	0.19***
Study 3 (cross-sectional t2)	−0.18***	−0.22***	−0.22***	−0.19***	0.22***	0.17***
Study 3 (comp. predicting t2)	−0.16***	−0.18***	−0.16***	−0.19***	0.19***	0.16***
Study 4	−0.14***	−0.23***	−0.17***	−0.20***	0.22***	0.10
**Metacognitions worries**						
Study 3 (cross-sectional t1)	0.29***	0.23***	0.26***	0.24***	−0.32***	−0.14***
Study 3 (cross-sectional t2)	0.29***	0.19***	0.28***	0.16***	−0.32***	−0.09
Study 3 (comp. predicting t2)	0.25***	0.19***	0.21***	0.18***	−0.29***	−0.09
**Brooding Study 5**	0.24***	0.13***	0.25***	0.06	−0.19***	−0.03
**Post-migration Stress Study 7**	0.10***	0.23***	–	–	–	–
**Self-efficacy Study 7**	−1.17***	−0.93***	–	–	–	–

### Associations with mental health predictor variables

For self-esteem, estimates ranged from −0.03 for temporal comparison discrepancy to 0.24 for social comparison affective impact. In all models (i.e., 18 comparisons of estimates), two estimates were not significant (temporal comparison discrepancy and temporal comparison affective impact).

For metacognitions about worries, estimates ranged from −0.03 for temporal comparison affective impact to −0.32 for social comparison affective impact. In the 12 models, four estimates were non-significant (comparison discrepancy (1x), and affective impact (3x)). Here, social comparisons were more strongly associated with metacognitions than temporal comparisons. For brooding, estimates ranged from −0.03 for temporal comparison affective impact to 0.25 for social comparison discrepancy. Out of the 6 estimates, (i.e., 3 comparisons of correlations), two were non-significant (i.e., temporal comparison discrepancy and temporal comparison affective impact). For postmigration stress, in the refugee sample (Study 7), the estimates for both social and temporal comparison frequency contributed unique variance to the model.

## Discussion

Across seven studies, both social and temporal comparisons were related to the assessed mental health outcomes and mental health predictors. Additionally, these comparison types showed mostly similar associations with our outcomes. In some instances, however, social comparisons exhibited slightly stronger associations with mental health variables relative to temporal comparisons.

Specifically, both social and temporal comparisons showed relationships with depression, anxiety, PTSD, psychological well-being, life satisfaction, self-esteem, metacognitions about worry, and rumination, demonstrating predominantly moderate effect sizes. As they were simultaneously included in the regression models, they contributed unique variance to the outcomes beyond the other comparison type. This aligns with previous research on social comparison and comparative thinking more generally (Anson et al., 2015; Goodman et al., 2021; McCarthy & Morina, 2020; Morina et al., 2023; Schlechter, Hoppen et al., 2024, 2023), extending the associations to temporal comparisons (Hoppen et al., 2020; Zell & Alicke, 2010). The observed associations highlight the significance of comparison discrepancy and comparison affective impact as crucial process components in the comparison process (Albert, 1977; Festinger, 1954; Morina, 2021). The findings were consistently found across seven distinct studies.In some instances, the social comparison contributed unique variance, while temporal comparisons did contribute unique variance beyond social comparison, which accounted for comparison frequency, discrepancy, and affective impact. These findings corroborate experimental research indicating a pronounced reliance on social comparison over temporal comparison (Van Yperen & Leander, 2014). This suggests an important role of social comparisons on mental health outcomes, which may be attributable to social comparison being a salient form of self-evaluation (Festinger, 1954; Helgeson & Mickelson, 1995). Social comparisons are highly relevant in daily life (Arigo et al., 2020) and contribute to shaping self-perceptions within social contexts. However, temporal comparisons still displayed moderate, consistent and in the majority of cases similarly strong associations with mental health outcomes as social comparisons. Altogether, caution is warranted in overinterpreting these differential findings, as future research may unveil stronger associations among temporal (vs. social) comparisons and other mental health outcomes not assessed in our study. Potentially, the association between social and temporal comparisons with well-being outcomes depends on context variables. For example, in a study with individuals exposed to a recent vehicle-ramming attack (Morina, 2020), upward (i.e., aversive) past-temporal comparisons showed a stronger correlation with PTSD symptoms (*r* = 0.44) than upward social comparisons (*r* = 0.26). In that study, upward temporal comparisons were also more frequently reported than upward social comparisons, suggesting that shortly after traumatic events temporal comparisons become more prominent. Crucially, many of the current associations indicate an equally robust and strong relationship of these comparison standards.

### Implications

Our findings clearly suggest that both social and temporal comparisons play an important role in well-being. The differences in correlations in the present work were insufficient to conclude that one type of comparison is generally more relevant than the other type. Future research needs to examine potential interactions between different types of comparisons (Wilson & Ross, 2000). This should ideally be examined in experimental designs by disentangling social or temporal comparison to isolate the respective impact of social and temporal comparisons. Besides, future research needs to shed light on motives and mechanisms underlying social and temporal comparisons to help identify dysfunctional comparison habits and responses (Helgeson & Mickelson, 1995). Additionally, future research needs to examine how social and temporal comparisons influence each other over time, as people frequently use social comparisons to evaluate their well-being, while evaluating potential changes over time via temporal comparisons (Zell & Alicke, 2010).

While there is need for experimental and intervention studies before our findings can be translated into practices, our findings point to the general need to focus on social and temporal comparisons in individuals with mental health complaints (Arigo et al., 2024). Potential psychological interventions can integrate cognitive restructuring techniques to challenge and modify maladaptive thought patterns associated with aversive social and temporal comparisons, by focusing not only on comparison frequency, but also discrepancy and affective impact. Psychoeducation about social and temporal comparative thinking may help individuals to recognize and manage the emotional consequences of comparison processes.

### Limitations

A first limitation of this project is the absence of pre-registration and the reliance on secondary data analyses. Second, six of the seven studies were cross-sectional. Future research should prioritize experimental designs and Ecological Momentary Assessment studies to provide a more robust understanding of the temporal and causal effects (Arigo et al., 2020; Schlechter et al., 2025). Third, the reliance on data from Prolific and social media channels restricts the generalizability of our results. Specifically, our samples consisted of young adults with mean ages ranging from 25 to 32 years and in all but one study we relied on an online pool of potential participants residing OECD countries. While we included one sample with Syrian refugees, additional cultural differences in social norms, values, and the significance of temporal comparisons may influence the way individuals engage in and are affected by comparative thinking. Fourth, methodological differences between the studies may have introduced unknown biases in the results. Fifth, we investigated mental health constructs in subclinical or non-clinical samples, necessitating more data from clinical populations (McCarthy & Morina, 2020). A further limitation of the study is the lower internal consistency of the temporal comparison subscales relative to the social comparison subscales, which suggests greater potential for measurement error in the temporal comparison items. This may be an alternative explanation for the slightly stronger effects observed for the social comparison subscales, as higher reliability can lead to more robust associations.

### Conclusion

Findings from seven studies suggest that social and temporal comparisons represent transdiagnostic factors in the context of mental health. Both comparison types exhibited broadly comparable associations with mental health outcomes. Further investigation of the role of both social and temporal comparison in mental health is clearly warranted.

## Data availability statement

The data and R code used in this study are openly available: https://osf.io/nxwjh/?view_only=5484a0ad75df482eb428355fbf197634.

## Declaration of competing interest

The authors declare that they have no competing interests.

## Appendix Supplementary materials

Image, application 1
