# Supplementary material for: Comparing comparisons: A transdiagnostic investigation of social and temporal comparisons and their differential effects on mental health outcomes and well-being
Source: Int J Clin Health Psychol. 2025 Jul 21;25(3):100611. doi: 10.1016/j.ijchp.2025.100611 (PMC12305210; doi:10.1016/j.ijchp.2025.100611)
Supplement: Supplementary file 1 [file mmc1.docx]

**Supplemental Materials for:**

**Comparing Comparisons: A Transdiagnostic Investigation of Social and Temporal Comparisons and their Differential Effects on Mental Health Outcomes and Well-being**

**Short Title:** Social and Temporal Comparisons

**Disclosure statement**

The authors declare that they have no competing interests.

**Data availability statement**

The data and R code used in this study are openly available: <https://osf.io/nxwjh/?view_only=5484a0ad75df482eb428355fbf197634>

**Acknowledgements**

None

| **Table s1**  *Skewness and Kurtosis of all constructs* | | | | | | | |  |
| --- | --- | --- | --- | --- | --- | --- | --- | --- |
|  | Study 1  (*N* = 1,121) | Study 2  (*N* = 550) | Study 3 T1  (*N* = 1,084) | Study 3 T2  (*N* = 942) | Study 4  (*N* = 306) | Study 5  (*N* = 500) | Study 6  (*N* = 596) | Study 7  (*N* = 991) |
| **Descriptive Statistics** | *Skew/Kurtosis* | *Skew/Kurtosis* | *Skew/Kurtosis* | *Skew/Kurtosis* | *Skew/Kurtosis* | *Skew/Kurtosis* | *Skew/Kurtosis* | *Skew/Kurtosis* |
| *Comparison Process* |  |  |  |  |  |  |  |  |
| Frequency Social | -0.24/-0.78 | -0.11/-1.02 | -0.10/-1.03 | -0.10/-1.03 | -0.11/-0.79 | 0.32/-1.15 | -0.46/-0.44 | 0.46/-0.93 |
| Frequency Temporal | 0.11/-0.78 | 0.09/-0.95 | 0.22/-0.93 | 0.22/-0.93 | 0.24/-0.85 | 0.43/-0.41 | 0.11/-0.48 | 0.29/-0.58 |
| Discrepancy Social | -0.50/-0.47 | -0.25/-0.97 | -0.39/-0.87 | -0.39/-0.87 | -0.37/-0.50 | -0.08/-1.23 | - | - |
| Discrepancy Temporal | -0.10/-0.65 | -0.08/-0.89 | 0.11/-0.84 | 0.11/-0.84 | 0.13/-0.54 | -0.53/-014 | - | - |
| Affective Impact Social | -0.10/0.14 | -0.21/0.03 | -0.45/0.06 | -0.45/0.06 | -0.48/0.17 | 0.36/0.25 | 0.28/-0.16 | - |
| Affective Impact Temporal | -0.30/0.22 | -0.33/0.74 | -0.24/0.55 | -0.24/0.55 | -0.20/0.55 | 0.21/0.18 | 0.12/0.28 | - |
| *Mental Health Outcomes* |  |  |  |  |  |  |  |  |
| Depression | 0.55/-0.50 | 0.84/-0.27 | 0.62/-0.64 | 0.62/-0.64 | 0.70/-0.12 | 0.26/-0.67 | 0.49/-0.96 | - |
| Anxiety | - | 0.67/-0.65 | 0.53/-0.72 | 0.53/-0.72 | 0.68/-0.33 | - | 0.50/-0.96 | - |
| Psychological Well-Being* | 0.10/-0.45 | - | -0.06/-0.98 | -0.06/-0.98 | - | 0.13/-0.49 | 0.28/-1.04 | 0.26/-0.48 |
| PTSD | - | - | 0.24/-0.88 | 0.34/-0.78 | - | - | - | 0.05/-0.75 |
| *Predictor Variables* |  |  |  |  |  |  |  |  |
| Self-esteem | 0.13/-0.59 | - | -0.18/-0.55 | -0.18/-0.56 | -0.57/-0.44 | - | - | - |
| Metacognitions worries | - | - | 0.27/-0.99 | 0.26/-0.89 | - | - | - | - |
| Brooding | - | - | - | - | - | 0.11/-0.47 | - | - |
| Postmigration stressors | - | - | - | - | - | - | - | 0.08/-0.34 |
| Self-efficacy | - | - | - | - | - | - | - | -0.46/0.35 |

Note. *including life satisfaction.
